# Supplementary material for: The experience of cerebral palsy stigma amongst adults living in the UK and Ireland: A qualitative co-designed project
Source: PLoS One. 2025 Sep 9;20(9):e0331562. doi: 10.1371/journal.pone.0331562 (PMC12419618; doi:10.1371/journal.pone.0331562)
Supplement: Attachment — Submitted filename: review.docx [file pone.0331562.s001.docx]

The experience of cerebral palsy stigma amongst adults living in the UK and Ireland: A

qualitative co-designed project

General comments:

The article is interesting and well written. It helps understand the experiences of people with CP, and importantly was co-designed together with people who have CP. It adds to the literature and is a definitive asset to scholarly knowledge as well as providing practical implications.

Abstract:

The results as presented in the abstract are difficult to follow. The themes should all be presented in the same manner (for example theme 2: impact on participation). It is difficult to understand the flow between themes themselves and the complicated flow chart in words. The themes are presented most clearly in the conclusion (though the 5th is missing). Please rewrite the results in a clearer presentation.

Also, there is no mention of the quantitative results. This needs fixing (or removing the quantitative section)

Introduction:

The flow is good and explains the importance.

In the description of the ICF model, the order should be reversed in the sentence “to participate and take part in activities” in order to be in line with the model.

The social model of disability is not mentioned, despite that its message resonates in this article. It should be added to the introduction or the discussion (or both).

The paragraph describing CP says that the most common presenting feature includes fine motor, but gait is gross motor. It is also not correct to say that fine motor function leads to muscle spasticity nor balance nor posture issues. This whole section needs to be written more accurately.

The paragraph about CP also describes the GMFCS, which is very important, but the GMFCS is never mentioned in the article after this. It would have been useful in the demographic description of the participants, but I suppose if it was a self-reported then some people might not know their GMFCS level. If the GMFCS is not used in the research, it isn’t necessary to explain it. The heterogeneity can be described without it.

Paragraph starting “In order to tackle stigma”: the person-centred approach is very important for many reasons. Preventing stigma an important reason, but the approach should not be shrunk only to this. Please re-phrase this.

Methods:

It is not clear how the participants were recruited. Was the survey a link on the social media channels of charities, or was there an add asking people to contact the researchers and then they received a link to the survey or an email? If the survey was in on the site, how could people receive hard copies or phone help? There is no data as to how many of the respondents were people with CP and how many were proxies (care givers). This is very important as if all participants filled out the survey themselves, then the results only reflect this populations point of view.

PPI- please write out abbreviations in full the first time used

The section about resilience and wellbeing can be shortened and the results that only 40 answered should be in the results. This should also be explored briefly in the discussion.

Sources of stigma: It is hard to understand how “once in life” can be put in the same category as “multiple times”.

It would be useful to have the survey in an appendix.

Results:

Table 1:

Each descriptive category has a different N which is vey confusing. They range from 76 (education) to 86 (marital status). If this is the case, then write the N in the first column with the category.

Athetotic is a form of dystonic CP. Hemiplegia is a description of where there is tone (half the body versus diplegia ....) whereas the other descriptions are of the type of tone. Most people with hemiplegia are spastic. Is the “other” mobility ais a scooter? The table needs more work.

In the written description of the demographics most data is presented to one decimal except 40% using wheelchair. It needs to be consistent.

Stigma questions: consider rephasing: Participant (n= 41- 47) reported that the sources of stigmatisation or discrimination was most frequently public.....

Please explain how the data presented in this paragraph was calculated as it doesn’t fit with the data in Table 2. For example, 89.1% (public): 80% + 11.1% doesn't add up to 89.1%. This is the same with other data taken from table 2. The numbers don’t seem to fit the data in the table, so please explain how this was done.

Table 2: please add the N to each row in the first column, then there is no need for the explanation at the end.

Stigma questions: Qualitative:

Please end quotation marks around the phrase rigid stereotypes

Please try to write the paragraph about the five themes in a clearer manner and write all themes in the same method (sometimes the theme itself is in parenthesis, sometimes the number is also and sometimes just the number). It is hard to follow. In the thematic map, the arrow between theme 1 and 2 should be uni-directional. Only 1 to 2 and not back also, if I understood correctly. Also, the sub-themes should be stated clearly (i.e. write subthemes at the top of the column or in each circle).

Rigid Stereotypes:

The sentence “In some cases, this misunderstanding that people with CP are not able to function could lead to unsolicited offers of help which were not always required” does not seem to fit the theme. This is not the stereotype, rather peoples’ actions.

Discussion:

The summery of the themes in the first paragraph is very well done. The clearest in the article.

There doesn’t seem to be any discussion of the quantitative data. Despite that this data is weaker than the qualitative data, it should be discussed or removed from the article. Please state the importance of data found from table 2, both in the discussion and conclusion.

The inclusion of other people with other disabilities or other people in wheelchairs is very interesting.

Limitations:

The fact that the people who responded to the survey were all literate, and many with post-secondary education, should be included in the limitations as there is little representation of the people with CP in the lower end of the spectrum (and we don’t know if any proxies participated to give information for them). The lack of GMFCS is also a limitation. The wide spectrum of people with CP is discussed, but there is little information about the representation of the people with CP who are lower functioning.

Implications:

Please explain how positive impact of seeking support from peers with CP will tackle stigma.

Conclusions:

The 5th theme is missing in the conclusion (which is a pity as it is very interesting).

The sentence “Many of the ways in which stigma and discrimination present for this group are common experiences amongst people with different visible disabilities” does not seem to be a conclusion from the data of this study.

As stated at the start, the article is very interesting, well written and adds to knowledge in the field. The comments are relatively minor and changes will make this article a great asset.
